# Association between tooth loss and frailty among Chinese older adults: the mediating role of dietary diversity

**DOI:** 10.1186/s12877-023-04355-6

**Published:** 2023-10-17

**Authors:** Xin Xu, Yuan Zhao, Bei Wu, Yaolin Pei, Danan Gu

**Affiliations:** 1 Population Research Institute, Nanjing University of Posts and Telecommunications, Nanjing, China; 2 School of Geography, Nanjing Normal University, Nanjing, China; 3 Jiangsu Center for Collaborative Innovation in Geographical Information Resource Development and Application, Nanjing, China; 4 Rory Meyers College of Nursing, New York University, 433 First Avenue, 10010 New York, NY USA; 5 Independent Researcher, New York, USA

**Keywords:** Tooth loss, Frailty, Dietary diversity, Chinese older adults, Mediation, CLHLS

## Abstract

**Background:**

This study aimed to examine the association between tooth loss and frailty among Chinese older adults and the mediating role of dietary diversity in this association.

**Methods:**

Data from five waves of the Chinese Longitudinal Healthy Longevity Survey conducted between 2005 and 2018 were used. Path analyses were employed to assess both concurrent and cross-lagged relationships between tooth loss and frailty index while accounting for intrapersonal correlation. Furthermore, the mediation effect of dietary diversity was also examined.

**Results:**

In concurrent models, severe tooth loss was associated with frailty after adjusting for demographic characteristics (odds ratio [OR] = 1.82, p < 0.001). The OR of frailty for severe tooth loss was only slightly decreased to 1.74 (p < 0.001) when dietary diversity was added to the model and to 1.64 (p < 0.001) when socioeconomic status, family support, and healthy lifestyles were further adjusted. In the cross-lag or longitudinal models, the ORs were mildly or moderately reduced to 1.29, 1.27, and 1.23, respectively, yet remained statistically significant (p < 0.001 or p < 0.01). The mediation analyses showed that dietary diversity had some small yet significant effects on the relationship between tooth loss and frailty in both concurrent and longitudinal settings.

**Conclusions:**

This study improves current knowledge regarding the impact of tooth loss on frailty among Chinese older adults. Future intervention strategies designed to improve healthy diets may have preventive effects against the risk of frailty among Chinese older adults with severe tooth loss.

**Supplementary Information:**

The online version contains supplementary material available at 10.1186/s12877-023-04355-6.

## Introduction

Frailty refers to an age-related decline in function that results in an inability to perform independent daily living activities and to cope with external stressors [1]. It has been reported to be a predictor of adverse clinical outcomes, such as mortality [2], falls [3], and hospitalization [4]. As the proportion of older adults in China has progressively increased [5], frailty in later life has become a major public health concern and places a significant burden on individuals, families, and healthcare systems. Among the various operationalization definitions of frailty, the frailty index developed by Mitnitski et al. based on deficit accumulation is widely used in literature [6, 7]. This index includes deficits such as chronic disease conditions, psychological illnesses, cognitive impairment, and other symptoms. However, oral health, an important predictor of physical health in older adults [8], has been neglected in this index, a critical research gap this study aims to fill.

Poor oral health is disproportionately common in older adults [9, 10]. Studies have shown that tooth loss may increase the risk of frailty in older adults [11]. Having at least 20 teeth is essential for maintaining good oral function, and functional dentition is significantly associated with a lower risk of frailty [12]. However, most previous studies on the association between tooth loss and frailty used a cross-sectional design [13, 14]. Therefore, the dynamic relationship between tooth loss and frailty in longitudinal data remains understudied. Furthermore, understanding oral health and its connection with frailty is particularly important in the Chinese context, as the Chinese healthcare system has long ignored oral health. The different cultural norms and values around oral health in China compared with Western countries also contribute to differences in the cross-cultural understanding of oral health.

Dietary diversity refers to the number of different foods or food groups regularly consumed. This measure is a good indicator of dietary quality and is also related to nutritional status [15]. Previous evidence has demonstrated a strong link between tooth loss and nutrition [11]; that is, tooth loss affects diet in terms of food choice, eating behavior, and dietary intake; inadequate dietary intake which eventually leads to malnutrition, weight loss, and reduced muscle mass and body function [10, 16]. Thus, dietary diversity may mediate the relationship between tooth loss and frailty. As few studies have explored the mediating role of dietary diversity in the relationship between tooth loss and frailty among older Chinese, our study aimed to expand the literature and fill the gap regarding a mediator of this association. The specific research questions of this study were as follows: (1) Is tooth loss—an indicator of oral health—associated with frailty index in older Chinese? (2) Is this association mediated by dietary diversity?

## Methods

### Sample

This study used data from the 2005, 2008/2009 (briefly as 2008), 2011/2012 (briefly as 2011), 2014, and 2017/2019 (briefly as 2018) waves of the Chinese Longitudinal Healthy Longevity Survey (CLHLS). The CLHLS, initially launched in 1998, is an ongoing prospective longitudinal cohort study investigating determinants of health and longevity in mainland China (hereafter China) from a multi-disciplinary perspective. The CLHLS uses a multistage and stratified cluster-sampling design to collect extensive information from adults aged 65 or older in randomly selected half of all cities/counties in 22 of the 31 provinces in which Han Chinese account for the majority of the population. Since 2008, one county from Hainan Province (the 23rd province of CLHLS) was added to the sample. The sampled provinces represented approximately 85% of the Chinese population [17]. Additionally, the CLHLS aims to interview all centenarians in the sampled cities/counties. The age of each centenarian is confirmed from various information, including genealogy documents, birth certificates, family handbooks, and the ages of their children and siblings. For every three centenarians, the CLHLS identifies roughly four or five nearby (living in the same county/district) octogenarians, nonagenarians, or adults aged 65–79 of the same sex with previous designated age and urban-rural residence, and randomly interviews them [18]. In other words, CLHLS oversampled very old persons and men as well as, to some extent, rural older adults. The details of the CLHLS sampling design, response rate, attrition, and data quality have been extensively described elsewhere [17, 19].

The numbers of original respondents aged 65 years and older who participated in the 2005, 2008, 2011, 2014, and 2018 waves were 15,613, 16,563, 9,679, 7,107, and 15,728, respectively, which consists of the newly recruited participants 9,111 in 2008, 1,386 in 2011, 1,099 in 2014, and 12,365 in 2018. The large reduction in the new sample recruitment in 2011 and 2014 waves were due to the shortage of research fund. We pooled these five waves together. Overall, there are 64,690 observations which are nested within 39,574 participants in our pooled cross-section or concurrent study. The detailed sample distribution by survey year, survival status, and number of interviews is listed in Appendix (see Table A3 in Appendix).

We also examined the longitudinal/cross-lag association between toot loss and frailty. In longitudinal models, there are 25,116 observations between these five waves from 13,013 participants (see Table A3 in Appendix). The large reduction in the sample size in longitudinal analyses compared to the concurrent setting is due to the exclusion of all participants in 2018, high mortality among the samples (more than 50% respondents died in 2005–2018), and some losses to follow-up (more than 10% of the respondents) [19].

For variables with missing values, we used multiple imputations with 100 imputations that drew on all relevant variables included in Table 1, except for the outcome variable to avoid biases associated with missing values [20]. All statistical analyses were performed using STATA 14.1.

**Table 1:** Baseline characteristics, CLHLS 2005–2018

Characteristics	% ^a^	Score of frailty index	p
Total number of participants	39,574	0.41	
Frailty status			
Non-frail	59.2		
Frail	40.8		
Tooth loss			
<=12 (remaining ≥ 20 teeth)	18.2	0.14	p < 0.001
13–32 (remaining 0–19 teeth)	80.0	0.48	
Mean age (years)	86.9		
Age groups			
Young-old (aged 65–79 years)	29.0	0.08	p < 0.001
Oldest-old (aged 80 + years)	71.0	0.54	
Gender			
Female	58.0	0.49	p < 0.001
Male	42.0	0.29	
Residence			
Rural	54.7	0.39	p < 0.001
Urban	45.3	0.43	
Years of schooling			
0	59.9	0.51	p < 0.001
1+	40.1	0.26	
Economic independence			
No	69.6	0.49	p < 0.001
Yes	30.4	0.23	
Marital status			
Not married	66.1	0.53	p < 0.001
Married	33.9	0.18	
Proximate to children			
Low proximate with children	14.7	0.38	p < 0.001
High proximate with children	85.3	0.42	
Current smoking			
No	83.2	0.45	p < 0.001
Yes	16.8	0.22	
Regular exercising			
No	72.1	0.49	p < 0.001
Yes	27.9	0.20	
Mean dietary diversity score	4.49		
Wave			
2005	39.4	0.38	p < 0.001
2008/2009	23.0	0.46	
2011/2012	3.5	0.36	
2014	2.8	0.42	
2017/2019	31.3	0.41	

Note: (1) This table is for participants at their first interviews only (or the new participants at each wave) from 2005 to 2018. (2) a, except for the total number of participants, the mean age, and the mean dietary diversity score, all other figures refer to percentages. The percentage distribution was not weighted. The distribution from observations is similar to except for distribution by wave. The weighted distribution is also similar with exceptions for waves, ages. The added percentages may not be equal to 100% due to rounding. (3) The score of frailty index ranges from 0 to 1. (4) p values of significance test were from Pearson chi square tests, and p values from the Likelihood-ratio chi square test are the same. p values from the Cramer’s V test, the gamma test, and the Kendall’s tau-b test are all significant at p < 0.05

### Measures

#### Dependent Variables

Frailty was assessed according to the Canadian Study of Health and Aging-Frailty Index, which characterizes the variation in health among people of the same chronological age by assuming that aging results in an accumulation of deficits [6]. This approach was applied to older adults in China [21] and its validity was verified [22]. Following the practice of these studies [21, 22], we selected 38 indicators representing 8 dimensions of health: activities of daily living (ADLs), instrumental activities of daily living (IADLs), functional limitations, cognitive function, self-rated health, hearing and vision impairments, psychological distress, and chronic diseases.

- IADLs and ADLs were assessed by whether a respondent needed any assistance in performing eight independent living activities (visiting neighbors, shopping, cooking, washing clothing, walking, and lifting) and six basic daily activities (bathing, dressing, toileting, indoor transferring, eating, and incontinence).
- Functional limitations were assessed by the ability to perform the following five actions: putting their hand behind their neck, raising their arms upright, putting their hand behind their lower back, standing up from sitting in a chair, and picking up a book from the floor.
- Cognition function was assessed by a validated Chinese version of the Mini-Mental State Examination (MMSE), which has a total score of 30; respondents with a score of 23 or lower were considered cognitively impaired. The ADL, IADL, and MMSE scales in CLHLS are all derived from the original scales that are widely used in the literature [19].
- Self-rated health encompassed three items: current self-rated health, health status compared to one year ago, and interviewer-rated health.
- Psychological distress was assessed by whether respondents always/often experienced loneliness, uselessness, or fearfulness.
- Chronic diseases were measured according to whether a respondent had the following chronic conditions: hypertension, diabetes, tuberculosis, heart disease, cerebrovascular disease, bronchitis, cancer, arthritis, bedsores, gastric disease, or Parkinson’s disease.

Each deficit was coded 1 if present and 0 if absent. The frailty index score was then calculated by dividing the total number of deficits over the total number of possible deficits (38 indicators). Respondents with 9 or fewer deficits were considered nonfrail (Frailty index < 0.25), whereas those with 10 or more deficits (Frailty index ≥ 0.25) were considered frail [23].

Independent Variables.

The number of natural teeth was self-reported. Previous studies have shown that having at least 20 teeth represents acceptable oral health and functional dentition [12], whereas having less than 10 teeth is linked to a lower nutritional and functional status of older adults [24]. We divided tooth loss into two categories: severe tooth loss (12 missing teeth < tooth loss ≤ 32 missing teeth) and not-severe tooth loss (tooth loss ≤ 12 missing teeth) [24].

#### Mediator

The dietary diversity score (DDS) was assessed by a food frequency questionnaire related to seven major food groups: meat, fish, eggs, beans, sugar, garlic, and fresh vegetables. We recoded consumption of each food group as a dichotomous variable (1 = consume very often or almost every day, 0 = less frequent consumption). The DDS was then measured as the sum of the nine groups of food scores, ranging from 0 to 7, with a higher score indicating high dietary diversity [25]. Cereals and oil were not included in the DDS as almost all Chinese eat these two types of food every day [26]. Prior studies have shown that DDS is a simple and straightforward means of identifying individuals at high risk for mortality even for the oldest-old [25].

#### Covariates

To obtain robust results, we controlled many key confounders identified in the literature to be associated with frailty: demographic characteristics, socioeconomic status, family support, and healthy lifestyles. Demographic characteristics included age, gender (male = 1), and urban/rural residence (urban = 1). Socioeconomic status included education (one or more years of education = 1), and economic independence (primary financial source from retirement wages or work = 1). Family support included marital status (married = 1), and proximity to children (living in the same residence or village as children = 1). Lifestyle variables included current smoking status (yes = 1) and regular exercising (yes = 1).

### Analytical strategies

To examine the relationship between tooth loss and frailty, how this relationship is mediated by the dietary diversity, and how the relationship and the mediation were altered when other confounding factors were present, we employed two sets of analyses: one with no mediation and the other with mediation. The first set included three subsequent logit regression models after adjusting for intrapersonal correlation based on the GSEM command in Stata. In other words, we employed the multilevel analysis technique with observations as the first analytical unit and participants as the second analytical unit. In Model I, we examined the main effect of tooth loss on frailty after controlling for demographic characteristics. The reason for controlling these demographic factors is because they determined how the CLHLS samples were selected as abovementioned. In Model II, DDS was added to analyze whether the association between dietary diversity and frailty was altered in the presence of the dietary diversity. In Model III (or the full model), socioeconomic status, family support, and healthy lifestyles were further controlled to examine whether the relationship was modified by these confounders.

The three-model design strategy was further applied to both the concurrent (cross-sectional) and the cross-lagged (longitudinal) relationships between tooth loss and frailty. In cross-lagged relationship analysis, the outcome variable (i.e., frailty) was measured at Time (T + 1), whereas all other variables were measured at T.

#### Testing the mediation by dietary diversity

In the second set of analysis, which is the mediation analysis, we aimed to examine how dietary diversity mediated the relationship between tooth loss and frailty. The mediation analysis includes two structural equations: one logit model for the frailty equation and one linear model for the mediation equation. In the frailty equation we examined the direct effect of tooth loss and the dietary diversity on frailty, whereas in the mediation equation we examined the relationship between tooth loss and frailty via the dietary diversity. From these path analyses, we can estimate the direct, indirect (through dietary diversity), and total effect of tooth loss on frailty. Two sequential models were designed. Model I adjusted for demographics and Model II (or the full model) additionally adjusted for other covariates.

We also used the GSEM command in Stata to test the mediation or pathway by which older adults’ tooth loss affected their dietary diversity and ultimately their frailty after adjusting intrapersonal correlation given that many participants have more than one interviews due to its longitudinal nature of CLHLS. By doing so, we determined whether older adults’ tooth loss exerted its effects through the hypothesized pathway. Since the GSEM procedure does not produce goodness-of-fit metrics, we report the Akaike information criterion (AIC) and Bayesian information criterion (BIC) values for the model [27]. Like non-mediation analyses, we applied this modeling design to both the pooled cross-sectional data and the longitudinal data.

To test the robustness of our modeling results, we also treated frailty as a continuous variable (logarithm transformation after the score 0 was coded as 0.0001) and an ordinal variable (quartiles) in both analytical settings. Both these sensitivity analyses confirmed our current findings (see Appendixes).

## Results

### Sample characteristics

Table 1 presents the baseline characteristics (from the first interview) of CLHLS participants in the overall sample. We included a total of 39,574 participants. The mean score of frailty index (ranging from 0 to 1) for all participants was 0.41. Approximately 40.8% of the participants were categorized as frail during the study period. A total of 80.0% of participants had severe tooth loss. The mean age of these participants was 86.9 years (range: 65 to 120), and 71.0% of these participants were oldest-old aged 80 or older. More than half of the entire sample were female (58.0%), rural residents (54.7%), and those with no formal education 59.9%). About two-thirds of participants were unmarried. The majority of the participants were financially dependent (69.6%) and lived close to their children (85.3%).

### Regression analyses without mediation

As depicted the concurrent models (the left panel) in Table 2, severe tooth loss was significantly associated 82 per cent higher odds with frailty (OR = 1.82, *p* < 0.001) than non-severe tooth loss, after controlling for demographic characteristics. When controlling for diet diversity, OR of being frail for the severe tooth loss was only slightly changed (OR = 1.74, *p* < 0.001). Even after controlling for socioeconomic status, family support, and lifestyle measures (see Model III or the full model), this significant relationship still persisted (OR = 1.64, *p* < 0.001).

**Table 2:** Associations between tooth loss and frailty without mediation, CLHLS, 2005–2018

	Concurrent			Longitudinal		
	Mode I	Model II	Model III	Mode I	Model II	Model III
**Odds ratios for models of frailty** ^**a**^						
Severe tooth loss	1.82***	1.74***	1.64***	1.29***	1.27***	1.24**
Dietary diversity score (DDS)		0.84***	0.88***		0.95**	0.97*
Age	1.19***	1.19***	1.17***	1.20***	1.20***	1.19***
Male	0.51***	0.52***	0.73***	0.48***	0.49***	0.62***
Urban	1.30***	1.40***	1.64***	1.26***	1.28***	1.39***
1 + years of schooling			0.93			0.81**
Economic independence			0.94			0.90
Married			0.84***			0.91
High Proximate to children			0.99			1.01
Current smoking			0.51***			0.80**
Regular exercising			0.23***			0.73***
Wave 2008 (2005)	1.03	0.86***	0.85***	1.24***	1.18**	1.18**
Wave 2011 (2005)	1.35***	1.14**	1.18***	1.16*	1.11	1.13
Wave 2014 (2005)	1.37***	1.17**	1.09	1.74***	1.67***	1.65***
Wave 2018 (2005)	1.43***	1.23***	1.19***	NA ^b^	NA ^b^	NA ^b^
No. of observations	64,690	64,690	64,690	25,116	25,116	25,116
Number of individuals	39,574	39,574	39,574	13,013	13,013	13,013
-LL	31,492.5	31,278.1	30,086.2	12,315.6	12,310.2	12,271.3
AIC	63,004.9	62,578.1	60,206.3	24,649.2	24,640.4	24,574.6
BIC	63,095.7	62,678.0	60,360.6	24,722.4	24,721.7	24,704.7

Note: (1) a, Odds ratios were obtained from logistic regression using the -gsem- command. (2) b, NA, not applicable. In the longitudinal models, all independent variables were measured by one wave prior to the wave at which the outcome variable was measured. (3) ***p < 0.001; **p < 0.01; *p < 0.05

In the longitudinal models (the right panel in Table 2), these three odds ratios were mildly attenuated to 1.29, 1.27, and 1.24, respectively, but they were still statistically significant. These findings indicate that a higher level of tooth loss was associated with greater frailty among older Chinese, and that the concurrent association is more pronounced than the longitudinal association. Sensitivity analyses (Tables A1a and A1b in Appendix) show that the associations between tooth loss and frailty is still valid when frailty index was considered a continuous variable or an ordinal variable.

### Mediation analysis

Table 3 presents the results of the mediation analysis for the tooth loss and frailty with dietary diversity as the mediator. In the first part (top panel), the logit model, the direct associations between severe tooth loss and frailty and between DDS and frailty are the same as those Models II and III in Table 2. DDS was negatively associated with frailty. Specially, in the concurrent dataset, increasing one score in the dietary diversity index was associated with 16% lower odds (OR = exp (-0.171) = 0.84, p < 0.001) of being frail when demographics were adjusted for. In the full model when all study covariates were controlled, one score increase in DDS was associated with 12% lower odds (OR = exp (-0.132) = 0.88, p < 0.001). ORs for DDS were moderately attenuated in the longitudinal setting, but the association between DDS and frailty was still significant.

**Table 3:** Associations between tooth loss and frailty with mediation, CLHLS, 2005–2018

	Concurrent		Longitudinal	
	Model I	Model II	Model III	Model IV
**Models for frailty (logit model) (outcome variable: Frailty index)** ^**a**^				
Severe tooth loss	0.555***	0.494***	0.242***	0.213**
Dietary diversity score (DDS)	-0.171***	-0.132***	-0.046**	-0.031*
Age	0.174***	0.157***	0.182***	0.172***
Male	-0.657***	-0.319***	-0.716***	-0.482***
Urban	0.340***	0.494***	0.249***	0.331***
1 + years of schooling		-0.071		-0.215**
Economic independence		-0.066		-0.110
Married		-0.174***		-0.095
High Proximate to children		-0.009		0.013
Current smoking		0.667***		-0.229**
Current exercise		-1.467***		-0.314***
Wave 2008 (2005)	-0.150***	-0.165***	0.165**	0.168**
Wave 2011 (2005)	0.130**	0.168***	0.104	0.122*
Wave 2014 (2005)	0.161**	0.087	0.517***	0.503***
Wave 2018 (2005)	0.210***	0.173***	NA ^c^	NA ^c^
**Mediation equation (linear model) (outcome variable: Dietary diversity score)** ^**b**^				
Severe tooth loss	-0.304***	-0.238***	-0.263***	-0.211***
Age	-0.009***	0.003	-0.006***	0.005***
Male	0.182***	-0.039*	0.221***	-0.007
Urban	0.457***	-0.318***	0.439***	-0.314***
1 + years of schooling		0.326***		0.334***
Economic independence		0.344***		0.264***
Married		0.095***		0.112***
High Proximate to children		0.110***		0.077**
Current smoking		-0.029		-0.022
Regular exercising		0.348***		0.303***
Wave 2008 (2005)	-1.011***	-1.009***	-1.012***	-1.020***
Wave 2011 (2005)	-0.978***	-0.985***	-0.966***	-0.981***
Wave 2014 (2005)	-0.897***	-0.895***	-0.838***	-0.843***
Wave 2018 (2005)	-0.858***	-0.896***	NA ^c^	NA ^c^
Direct effect	0.555***	0.494***	0.242***	0.213***
Indirect effect	0.052***	0.031***	0.012***	0.007*
Total effect	0.607***	0.525***	0.254***	0.219***
% of indirect effect	8.6***	5.9***	4.7***	3.2***
No. of observations	64,690	64,690	25,116	25,116
N of individuals	39,574	39,574	13,013	13,013
-LL	154,364.5	152,298.3	59,539.2	59,212.9
AIC	308,773.0	304,664.6	119,118.5	118,489.8
BIC	308,972.7	304,973.2	119.281.1	118,750.0

Note: (1) a, Coefficients were obtained from logistic regression adjusting for intrapersonal correlation using the -gsem- command. (2) b, coefficients were obtained from linear regression adjusting for intrapersonal correlation using the -gsem- command. To ensure robustness, the error term in the logit model was modelled to be correlated with the error term in linear model. (3) c, NA, not applicable. In the longitudinal models, all independent variables were measured by one wave prior to the wave at which the outcome variable was measured. (4) ***p < 0.001; **p < 0.01; *p < 0.05

**Fig. 1:**
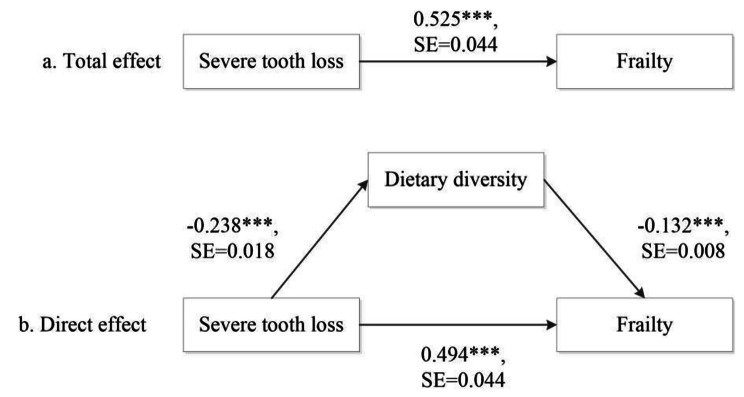
Mediation analysis for the concurrent relationship between tooth loss and frailty through dietary diversity Note: (1) The models controlled for age, sex, urban/rural residence, years of schooling, economic independence, marital status, proximity to children, current smoking status and regular exercising. (2) ***p < 0.001, **p < 0.01, *p < 0.001

**Fig. 2:**
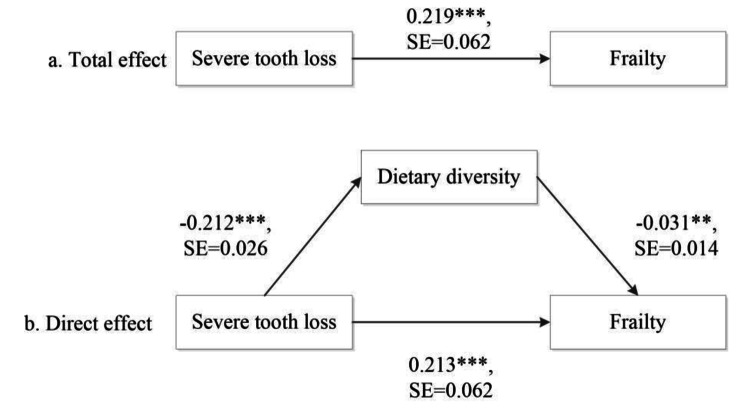
Mediation analysis for the longitudinal relationship between tooth loss and frailty through dietary diversity Note: (1) The models controlled for age, sex, urban/rural residence, years of schooling, economic independence, marital status, proximity to children, current smoking status, and regular exercising. (2) ***p < 0.001, **p < 0.01, *p < 0.001

In the second equation of the mediation model, that is the linear regression model, the severe tooth loss was negatively associated with DDS in both models and in both settings, although the coefficient was slightly lower in longitudinal models and in the full model. Overall, in the concurrent setting, the indirect effect of tooth loss on frailty through the dietary diversity was 0.052 (p < 0.001) when only demographics were controlled and 0.031 (p < 0.001) when all covariates were accounted for. In the longitudinal setting, these indirect effects were substantially reduced, but they were still statistically significant. Compared to its direct effect, the indirect effect of tooth loss through the dietary diversity is relatively small: 6–9% in the concurrent models and 3–5% in the longitudinal models). Figures 1 and 2 present the paths of the direct effect, the indirect effect, and the total effect in the full model under the concurrent and longitudinal designs. Sensity analyses in Appendix (Tables A2a and A2b and Figure A1) reveal that the direct effect, the indirect effect, and the total effect of tooth loss on frailty were still valid regardless of whether the frailty index was considered a continuous outcome or an ordinal outcome with exception for the indirect effect under the longitudinal setting when the frailty index was treated as a continuous variable.

## Discussion

To our knowledge, this is the first study to explore the relationship between tooth loss and frailty and the mediating effect of dietary diversity on this relationship. Using data from five waves of a nationally representative survey, the results of this study show that tooth loss is positively related to frailty among the aged populations. More importantly, the findings indicated that approximately 6–9% of this effect was mediated by dietary diversity in the concurrent models and 3–5% in the longitudinal models. Although the mediating effect through the dietary diversity is relatively small, these are still statistically significant, even after controlling for a wide set of confounders, thus revealing a pathway for interventions through dietary diversity to prevent frailty in older adults due to tooth loss.

Our results regarding tooth loss and frailty are consistent with studies conducted in other countries that demonstrated that tooth loss was associated with a greater likelihood of frailty [12, 24, 28–30). Previous studies have indicated that tooth loss reduces chewing performance and causes eating difficulties. People with severe tooth loss may change their dietary choices, such as reducing the intake of hard food (certain fruits, calcium, and proteins) and increasing the intake of softer, more energy dense foods, thereby increasing the intake of saturated fat and sugar and leading to malnutrition, frailty, and further dysfunction [10, 31]. An inappropriate or imbalanced diet could also lead to a decline in muscle mass, muscle strength, and physical function, and thereby contribute to frailty [32]. This pathway was consistent with the conclusions of our current study regarding the mediating role of dietary diversity.

The significant association between tooth loss and frailty may be explained by the following possible underlying mechanisms. First, dental conditions have psychosocial effects [12], and severe tooth loss can lead to facial deformities that affect language, social image, and self-esteem. These changes may limit people’s social interactions and social engagement. Reduction in social activity can lead to cognitive or mental disorders, both of which increase the risk of frailty [24]. Second, systemic conditions (e.g., diabetes and cardiovascular diseases) and oral inflammation may contribute to the link between tooth loss and frailty. Studies have revealed that adults with diabetes are at higher risk of experiencing tooth loss and edentulism than adults without diabetes [33–35]. Periodontitis and oral inflammation are the main causes of tooth loss in later life; these diseases could increase the risk of cerebrovascular disease and may lead to functional disability in older adults [29, 36].

Our finding of the significant role of dietary diversity in mediating the relationship between tooth loss and frailty is also interpretable. Studies have shown that dietary diversity improves survival and quality of life among older adults. For example, a higher DDS is associated with a reduced likelihood of consuming toxic food components, optimized telomere length, and better maintenance of muscle strength [37]. Thus, a lower DDS may lead to dietary deficiencies in essential and micronutrients, a particular concern for older adults. Specifically, the DDS used in our study included meat, fish, eggs, beans, salty vegetables, tea, garlic, and fresh vegetables. The best way to optimize health and longevity may be to consume low levels of protein at younger ages and then moderate to high levels of protein in old age [38]. However, insufficient protein intake is very common among older Chinese [39]. Thus, interventions targeting protein consumption are urgently needed as part of geriatric care to reduce undernutrition and improve the diet of older Chinese.

One strength of the present study is the use of a large sample size in an understudied context—the study included nearly 40, 000 older adults, recruited from 22 to 31 provinces in China to examine the association between tooth loss and frailty over a 13-year period. Among the participants, 71% were the oldest-old (aged 80 and above). This unique dataset provides the opportunity to examine a wide range of functional limitations as well as frailty. Second, the association between tooth loss and frailty has not been investigated among older Chinese; thus, our study filled this knowledge gap and provided insights into whether tooth loss constitutes the general pathogenesis of frailty. Third, our study used a longitudinal design with a long follow-up period from 2005 to 2018. The longitudinal analysis enabled us to examine tooth loss as a predictor of changes in frailty over a long period of time. Our result suggest that tooth loss may be an important indicator of frailty and could be included in the construction of a frailty index in future studies. Finally, using large-scale survey data, we examined dietary diversity as a mediator of the association between tooth loss and frailty, a relationship that has not been previously explored.

Some limitations should be noted. The number of remaining teeth was self-reported rather than determined with a clinical oral examination. Thus, the accuracy of self-reporting is a concern. However, several studies have shown that the number of self-reported teeth is a sound and reliable measure [40]. Future studies could use more sophisticated clinical oral health measures, such as dental caries and periodontal disease, to further examine this association. In addition, we lacked data on the timing of tooth loss and chewing ability, these variables may have specific effects on frailty in older Chinese. Furthermore, the dietary diversity score does not consider protein and calorie intake due to data unavailability. Additionally, individuals with severe tooth loss may experience reduced protein and energy intake, which can lead to physical frailty. Future studies are warranted to further examine the relationship in greater detail. Finally, about 15% of participants lost to follow-up were excluded from our analyses, which may introduce some biases in our models. The proportion of losses to follow-up in CLHLS is similar to major surveys of aging studies in other [41]. Fortunately, such biases should be mild because previous studies have shown that inclusion of those lost to follow-up by imputing these missing cases yielded comparable results with those from exclusion [42, 43].

## Conclusions

Our study found that severe tooth loss was closely associated with frailty among Chinese older adults. The observed association between tooth loss and frailty was significantly mediated by dietary diversity. Our findings expand the literature and indicate that tooth loss is an important indicator in screening for frailty among older Chinese. Improving oral health may be critical for maintaining the physical function of older adults in China, and interventions are warranted to improve dietary diversity and mitigate the adverse effects of tooth loss on frailty.

### Electronic supplementary material

Below is the link to the electronic supplementary material.

Supplementary Material 1

## Data Availability

The CLHLS datasets are publicly available at the National Archive of Computerized Data on Aging, University of Michigan (https://www.icpsr.umich.edu/web/NACDA/studies/37226). Researchers can obtain these data after submitting a data use agreement to the CLHLS team.

## Abbreviations

CLHLS: Chinese Longitudinal Healthy Longevity Survey
ADLs: Activities of daily living
IADLs: Instrumental activities of daily living
MMSE: Mini-Mental State Examination
DDS: Dietary diversity score
GSEM: Generalized structural equation modeling
ORs: Odds ratios
AIC: Akaike information criterion
BIC: Bayesian information criterion
