# Supplementary material for: Association between tooth loss and frailty among Chinese older adults: the mediating role of dietary diversity
Source: BMC Geriatr. 2023 Oct 17;23:668. doi: 10.1186/s12877-023-04355-6 (PMC10583397; doi:10.1186/s12877-023-04355-6)
Supplement: Supplementary file 1 — Supplementary Material 1 [file 12877_2023_4355_MOESM1_ESM.docx]

**Appendix**

Table A1. Associations between tooth loss and frailty without mediation, CLHLS, 2005-2018

A1a: Frailty index is a continuous variable

|  | Concurrent | | |  | Longitudinal | | |
| --- | --- | --- | --- | --- | --- | --- | --- |
|  | Mode I | Model II | Model III |  | Mode I | Model II | Model III |
| **Coefficient for models of frailty (linear model) (outcome variable: Frailty index) ^a^** | | | | | | | |
| Severe tooth loss | 0.208*** | 0.190*** | 0.173*** |  | 0.088*** | 0.082*** | 0.071*** |
| Dietary diversity score (DDS) |  | -0.061*** | -0.051*** |  |  | -0.023*** | -0.017** |
| Age | 0.050*** | 0.049*** | 0.044*** |  | 0.050*** | 0.050*** | 0.046*** |
| Male | -0.237*** | -0.226*** | -0.127*** |  | -0.245*** | -0.240*** | -0.156*** |
| Urban | 0.064*** | 0.093*** | 0.136*** |  | 0.090*** | 0.100*** | 0.126*** |
| 1+ years of schooling |  |  | -0.012 |  |  |  | -0.070*** |
| Economic independence |  |  | -0.056*** |  |  |  | -0.068*** |
| Married |  |  | -0.079*** |  |  |  | -0.062*** |
| High Proximate to children |  |  | -0.030** |  |  |  | -0.012 |
| Current smoking |  |  | -0.185*** |  |  |  | -0.08*** |
| Regular exercising |  |  | -0.303*** |  |  |  | -0.067*** |
| Wave 2008 (2005) | -0.002 | -0.064*** | -0.060*** |  | 0.083*** | 0.061*** | 0.067*** |
| Wave 2011 (2005) | 0.077*** | -0.016 | -0.028* |  | -0.038 | -0.016 | -0.023 |
| Wave 2014 (2005) | -0.052*** | -0.004 | -0.011 |  | 0.143*** | 0.123*** | 0.124**** |
| Wave 2018 (2005) | 0.118*** | 0.065*** | 0.066** |  | NA ^b^ | NA ^b^ | NA ^b^ |
|  |  |  |  |  |  |  |  |
| No. of observations | 64,690 | 64,690 | 64,690 |  | 25,116 | 25,116 | 25,116 |
| Number of individuals | 39,574 | 39,574 | 39,574 |  | 13,013 | 13,013 | 13,013 |
| -LL | 94324.6 | 94026.6 | 93,337.8 |  | 37,240 | 37,227 | 37,180.8 |
| AIC | 188,671.2 | 188,077.1 | 186,711.6 |  | 74,501.5 | 74,477.4 | 74,395.6 |
| BIC | 188,771.0 | 188,186.0 | 186,875.0 |  | 74,582.8 | 74,566.8 | 74,533.8 |

Note: (1) a, Coefficients were obtained from the linear regression using the -gsem- command. (2) b, NA, not applicable. In the longitudinal models, all independent variables were measured by one wave prior to the wave at which the outcome variable was measured. (3) ***p<0.001; **p<0.01; *p<0.05.

A1b. Frailty index was grouped as a quartile variable

|  | Concurrent | | |  | Longitudinal | | |
| --- | --- | --- | --- | --- | --- | --- | --- |
|  | Mode I | Model II | Model III |  | Mode I | Model II | Model III |
| **Odds ratios for models of frailty ^a^** | | | | | | | |
| Severe tooth loss | 1.609*** | 1.542*** | 1.488*** |  | 1.219*** | 1.210*** | 1.187*** |
| Dietary diversity score (DDS) |  | 0.854*** | 0.877*** |  |  | 0.966*** | 0.975* |
| Age | 1.159*** | 1.158*** | 1.145*** |  | 1.167*** | 1.166*** | 1.158*** |
| Male | 0.515*** | 0.538*** | 0.697*** |  | 0.497*** | 0.501*** | 0.598*** |
| Urban | 1.202*** | 1.288*** | 1.448*** |  | 1.224*** | 1.239*** | 1.309*** |
| 1+ years of schooling |  |  | 0.940* |  |  |  | 0.819*** |
| Economic independence |  |  | 0.925** |  |  |  | 0.908* |
| Married |  |  | 0.860*** |  |  |  | 0.943 |
| High Proximate to children |  |  | 0.930** |  |  |  | 0.967 |
| Current smoking |  |  | 0.555*** |  |  |  | 0.845*** |
| Regular exercising |  |  | 0.384*** |  |  |  | 0.836*** |
| Wave 2008 (2005) | 1.007 | 0.857*** | 0.857*** |  | 1.251*** | 1.206*** | 1.215** |
| Wave 2011 (2005) | 1.292*** | 1.106** | 1.141*** |  | 1.179*** | 1.137** | 1.151** |
| Wave 2014 (2005) | 1.252*** | 1.088* | 1.053 |  | 1.740*** | 1.683*** | 1.682*** |
| Wave 2018 (2005) | 1.442*** | 1.262*** | 1.249*** |  | NA ^b^ | NA ^b^ | NA ^b^ |
|  |  |  |  |  |  |  |  |
| No. of observations | 64,690 | 64,690 | 64,690 |  | 25,116 | 25,116 | 25,116 |
| Number of individuals | 39,574 | 39,574 | 39,574 |  | 13,013 | 13,013 | 13,013 |
| -LL | 73,679.4 | 73,323.3 | 72,156.9 |  | 29,027.5 | 29,021.3 | 28,985.8 |
| AIC | 147,382.7 | 146,673.6 | 144,351.7 |  | 58,076.9 | 58,066.7 | 58,007.7 |
| BIC | 147,491.7 | 146,790.6 | 144,524.2 |  | 58,166.3 | 58,164.3 | 58,154.0 |

Note: (1) a, Odds ratios were obtained from ordered logit regression using the -gsem- command. (2) b, NA, not applicable. In the longitudinal models, all independent variables were measured by one wave prior to the wave at which the outcome variable was measured. (3) ***p<0.001; **p<0.01; *p<0.05.

Table A2. Associations between tooth loss and frailty with mediation, CLHLS, 2005-2018

A2a: Frailty index is a continuous variable

|  | Concurrent | |  | Longitudinal | |
| --- | --- | --- | --- | --- | --- |
|  | Model I | Model II |  | Model I | Model II |
| **Models for frailty (linear model) (outcome variable: frailty index) ^a^** | | | | | |
| Severe tooth loss | 0.193*** | 0.174*** |  | 0.089*** | 0.075*** |
| Dietary diversity score (DDS) | -0.049*** | -0.046*** |  | -0.010 | 0.007 |
| Age | 0.049*** | 0.044*** |  | 0.050*** | 0.046*** |
| Male | -0.228*** | -0.127*** |  | -0.247*** | -0.157*** |
| Urban | 0.091*** | 0.135*** |  | 0.097*** | 0.124*** |
| 1+ years of schooling |  | -0.014 |  |  | -0.079*** |
| Economic independence |  | -0.057*** |  |  | -0.068*** |
| Married |  | -0.079*** |  |  | -0.064*** |
| High Proximate to children |  | -0.031** |  |  | -0.013 |
| Current smoking |  | -0.185*** |  |  | -0.081*** |
| Current exercise |  | -0.304*** |  |  | -0.070*** |
| Wave 2008 (2005) | -0.051*** | -0.055*** |  | 0.095*** | 0.093*** |
| Wave 2011 (2005) | 0.029* | 0.033* |  | 0.049** | 0.049** |
| Wave 2014 (2005) | 0.008** | -0.007 |  | 0.154*** | 0.149*** |
| Wave 2018 (2005) | 0.076*** | 0.070*** |  | NA ^c^ | NA ^c^ |
|  |  |  |  |  |  |
| **Mediation equation (linear model) (outcome variable: dietary diversity score) ^b^** | | | | | |
| Severe tooth loss | -0.303*** | -0.237*** |  | -0.253*** | -0.204*** |
| Age | -0.009*** | 0.003*** |  | -0.006*** | 0.004** |
| Male | 0.181*** | -0.039* |  | 0.218*** | -0.007 |
| Urban | 0.459*** | 0.319*** |  | 0.441*** | 0.317*** |
| 1+ years of schooling |  | 0.326*** |  |  | 0.334*** |
| Economic independence |  | 0.344*** |  |  | 0.259*** |
| Married |  | 0.095*** |  |  | 0.112*** |
| High Proximate to children |  | 0.109*** |  |  | 0.075** |
| Current smoking |  | -0.029 |  |  | -0.025 |
| Current exercise |  | 0.347*** |  |  | 0.297*** |
| Wave 2008 (2005) | -1.012*** | -1.010*** |  | -1.019*** | -1.026*** |
| Wave 2011 (2005) | -0.984*** | -0.987*** |  | -0.983*** | -0.995*** |
| Wave 2014 (2005) | -0.905*** | -0.898*** |  | -0.866*** | -0.865*** |
| Wave 2018 (2005) | -0.862*** | -0.898*** |  | NA ^c^ | NA ^c^ |
|  |  |  |  |  |  |
| Direct effect | 0.193*** | 0.174*** |  | 0.089*** | 0.075*** |
| Indirect effect | 0.015*** | 0.011*** |  | -0.003 | -0.002 |
| Total effect | 0.208*** | 0.185*** |  | 0.086*** | 0.074*** |
| % of indirect effect | 7.2*** | 5.9*** |  | -3.5 | -2.7 |
|  |  |  |  |  |  |
| No. of observations | 64,690 | 64,690 |  | 25,116 | 25,116 |
| N of individuals | 39,574 | 39,574 |  | 13,013 | 13,013 |
| -LL | 217,102.3 | 215,548.8 |  | 84,422.5 | 84,102.3 |
| AIC | 434,252.5 | 431,169.5 |  | 168,889.0 | 168,272.6 |
| BIC | 434,470.4 | 431,496.3 |  | 169,067.9 | 168,549.0 |

Note: (1) a, Coefficients were obtained from logistic regression adjusting for intrapersonal correlation using the -gsem- command. (2) b, coefficients were obtained from linear regression adjusting for intrapersonal correlation using the -gsem- command. To ensure robustness, the error term in the logit model was modelled to be correlated with the error term in linear model. (3) c, NA, not applicable. In the longitudinal models, all independent variables were measured by one wave prior to the wave at which the outcome variable was measured. (4) ***p<0.001; **p<0.01; *p<0.05.

A2b. Frailty index was grouped as a quartile variable

|  | Concurrent | |  | Longitudinal | |
| --- | --- | --- | --- | --- | --- |
|  | Model I | Model II |  | Model I | Model II |
| **Models for frailty (ologit model) (outcome variable: frailty index) ^a^** | | | | | |
| Severe tooth loss | 0.408*** | 0.371*** |  | 0.143** | 0.121** |
| Dietary diversity score (DDS) | -0.393*** | -0.374*** |  | -0.216*** | -0.212*** |
| Age | 0.155*** | 0.145*** |  | 0.165*** | 0.160*** |
| Male | -0.640*** | -0.390*** |  | -0.704*** | -0.563*** |
| Urban | 0.281*** | 0.401*** |  | 0.201*** | 0.262*** |
| 1+ years of schooling |  | 0.028 |  |  | -0.138* |
| Economic independence |  | -0.055 |  |  | -0.091* |
| Married |  | -0.143*** |  |  | -0.037 |
| High Proximate to children |  | -0.052 |  |  | -0.031 |
| Current smoking |  | -0.634*** |  |  | -0.159** |
| Current exercise |  | -0.951*** |  |  | -0.151*** |
| Wave 2008 (2005) | -0.384*** | -0.398*** |  | 0.031 | 0.031 |
| Wave 2011 (2005) | -0.073** | -0.053 |  | 0.009 | 0.010 |
| Wave 2014 (2005) | -0.063 | -0.109** |  | 0.463*** | 0.451*** |
| Wave 2018 (2005) | 0.083** | 0.053 |  | NA ^c^ | NA ^c^ |
|  |  |  |  |  |  |
| **Mediation equation (linear model) (outcome variable: dietary diversity score) ^b^** | | | | | |
| Severe tooth loss | -0.308*** | -0.242*** |  | -0.288*** | -0.238*** |
| Age | -0.007*** | 0.004** |  | -0.004** | 0.007* |
| Male | 0.190*** | -0.034 |  | 0.226*** | -0.013 |
| Urban | 0.419*** | -0.277*** |  | 0.401*** | -0.273*** |
| 1+ years of schooling |  | 0.335*** |  |  | 0.340*** |
| Economic independence |  | 0.326*** |  |  | 0.267*** |
| Married |  | 0.089*** |  |  | 0.114*** |
| High Proximate to children |  | 0.118*** |  |  | 0.075** |
| Current smoking |  | 0.027 |  |  | -0.001 |
| Current exercise |  | 0.370*** |  |  | 0.322*** |
| Wave 2008 (2005) | -0.996*** | -0.995*** |  | -0.993*** | -1.000*** |
| Wave 2011 (2005) | -0.924*** | -0.928*** |  | -0.909*** | -0.924*** |
| Wave 2014 (2005) | -0.824*** | -0.817*** |  | -0.751*** | -0.750*** |
| Wave 2018 (2005) | -0.816*** | -0.851*** |  | NA ^c^ | NA ^c^ |
|  |  |  |  |  |  |
| Direct effect | 0.408*** | 0.371*** |  | 0.143*** | 0.121* |
| Indirect effect | 0.121*** | 0.091*** |  | 0.062*** | 0.050*** |
| Total effect | 0.529*** | 0.462*** |  | 0.205*** | 0.171*** |
| % of indirect effect | 22.9*** | 19.7*** |  | 30.2*** | 29.2*** |
|  |  |  |  |  |  |
| No. of observations | 64,690 | 64,690 |  | 25,116 | 25,116 |
| N of individuals | 39,574 | 39,574 |  | 13,013 | 13,013 |
| -LL | 197,468.6 | 195,371.6 |  | 76,836.9 | 76,497.9 |
| AIC | 394,985.2 | 390,815.2 |  | 153,717.9 | 153,063.9 |
| BIC | 395,203.1 | 391,141.9 |  | 153,896.8 | 153,340.3 |

Note: (1) a, Coefficients were obtained from logistic regression adjusting for intrapersonal correlation using the -gsem- command. (2) b, coefficients were obtained from linear regression adjusting for intrapersonal correlation using the -gsem- command. To ensure robustness, the error term in the logit model was modelled to be correlated with the error term in linear model. (3) c, NA, not applicable. In the longitudinal models, all independent variables were measured by one wave prior to the wave at which the outcome variable was measured. (4) ***p<0.001; **p<0.01; *p<0.05.

Figure A1. The concurrent and longitudinal relationships between tooth loss and frailty with the dietary diversity as the mediator when frailty was considered a continuous or an ordinal variable

(I). Frailty index is a continuous variable (Concurrent relationship)


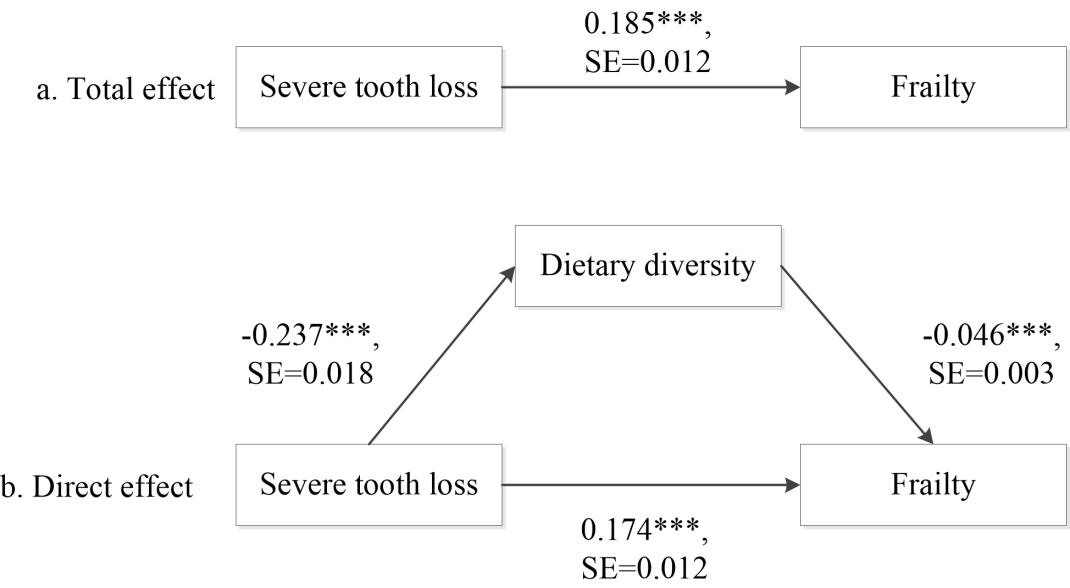


(II). Frailty index is a continuous variable (Longitudinal relationship)


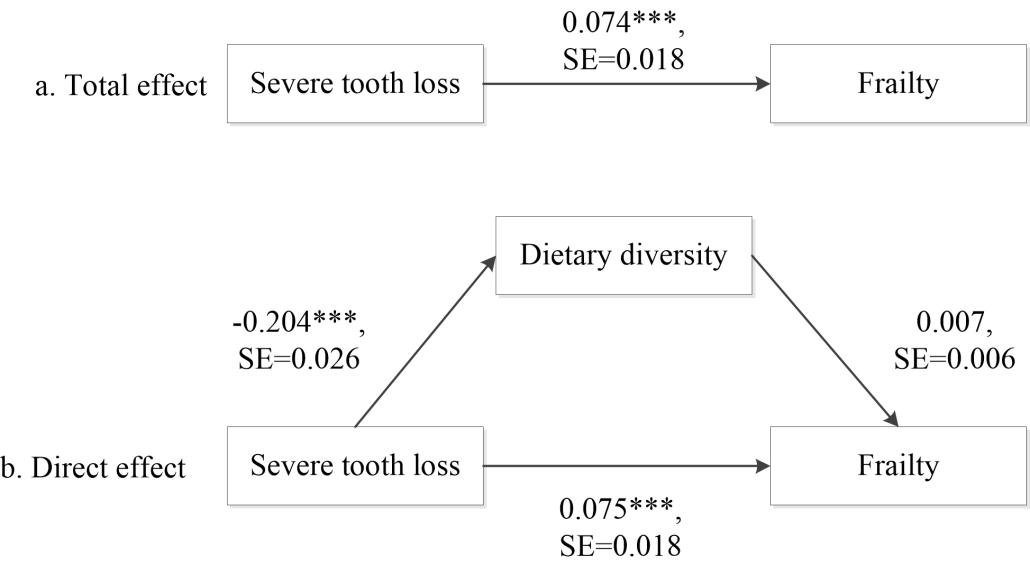


(Ⅲ). Frailty index was grouped as a quartile variable (Concurrent relationship)


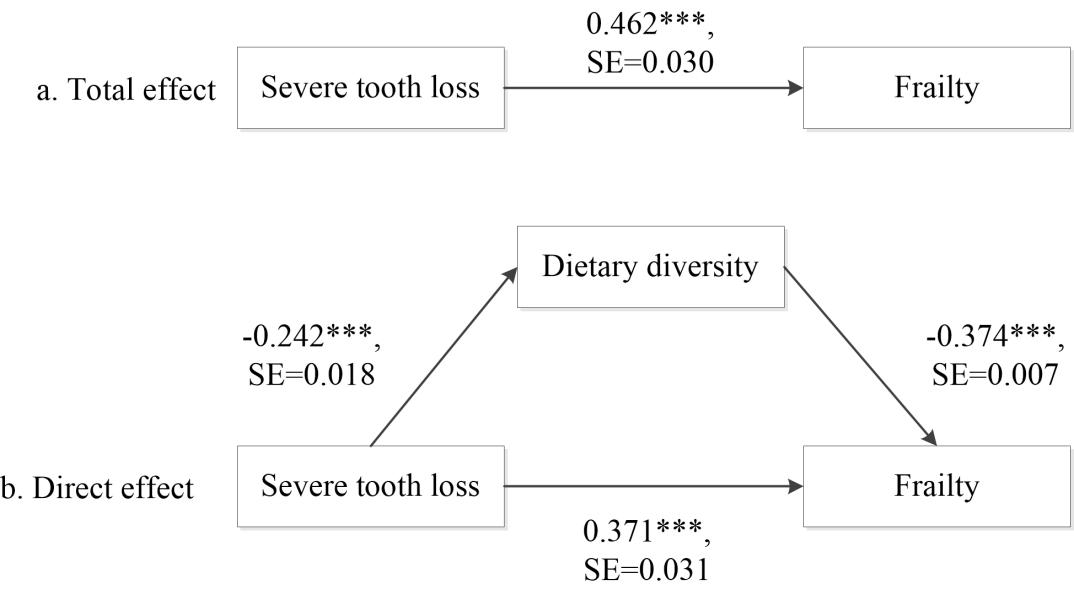


(Ⅳ). Frailty index was grouped as a quartile variable (Longitudinal relationship)


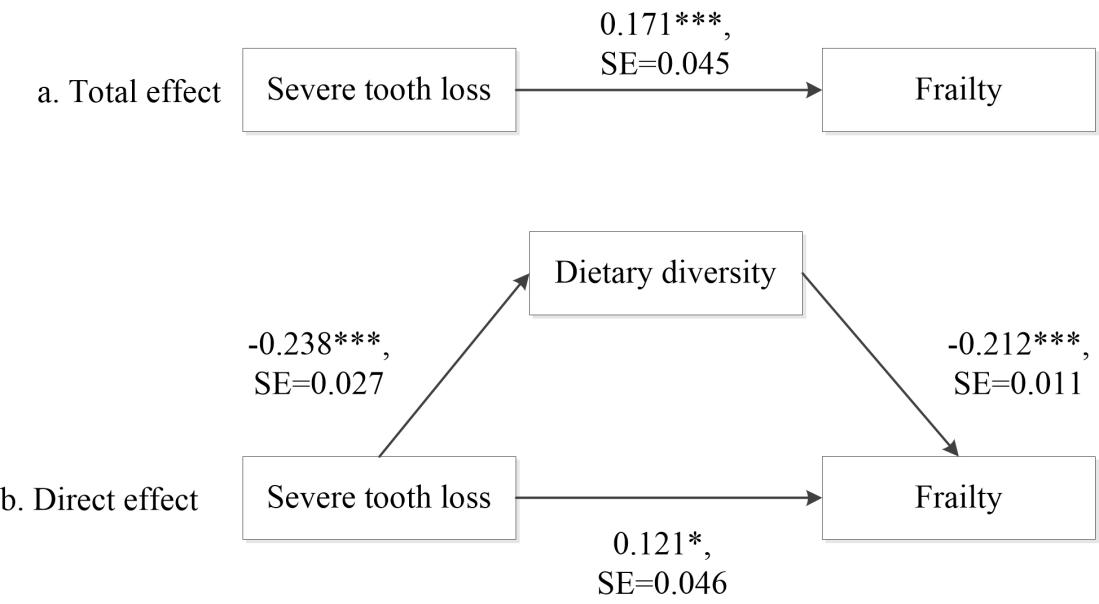


Note: (1) The models controlled from age, sex, urban/rural residence, years of schooling, economic independence, marital status, proximity to children, current smoking status, and regular exercising. (2) ***p<0.001, **p<0.01, *p<0.001.

Table A3. Sample distribution by survival status, survey year, and number of interviews, CLHLS, 2005-2018

|  | **2005** | **2008** | **2011** | **2014** | **Total** |
| --- | --- | --- | --- | --- | --- |
| **Survivors** |  |  |  |  |  |
| 1 interview | 7,453 | 4,119 | 901 | 540 | 13,013 |
| 2 interviews |  | 4,182 | 2,390 | 473 | 7,045 |
| 3 interviews |  |  | 2,784 | 950 | 3,734 |
| 4 interviews |  |  |  | 1,324 | 1,324 |
| Total | 7,453 | 8,301 | 6,075 | 3,287 | 25,116 |
|  |  |  |  |  |  |
|  | **2005-2008** | **2008-2011** | **2011-2014** | **2014-2018** | **Total** |
| **Deceased** |  |  |  |  |  |
| 1 interview | 5,222 | 3,371 | 294 | 389 | 9,276 |
| 2 interviews |  | 2,251 | 1,398 | 304 | 3,953 |
| 3 interviews |  |  | 1,182 | 860 | 2,042 |
| 4 interviews |  |  |  | 756 | 756 |
| Total | 5,222 | 5,622 | 2,874 | 2,309 | 16,027 |
| **Lost to follow-up** |  |  |  |  |  |
| 1 interview | 2,938 | 1,621 | 191 | 170 | 4,920 |
| 2 interviews |  | 1,019 | 327 | 98 | 1,444 |
| 3 interviews |  |  | 212 | 547 | 759 |
| 4 interviews |  |  |  | 696 | 696 |
| Total | 2,938 | 2,640 | 730 | 1,511 | 7,819 |
|  |  |  |  |  |  |
|  | **2005** | **2008** | **2011** | **2014** | **Total** |
| **Total** |  |  |  |  |  |
| 1 interview | 15,613 | 9,111 | 1,386 | 1,099 | 27,209 |
| 2 interviews |  | 7,452 | 4,115 | 875 | 12,442 |
| 3 interviews |  |  | 4,178 | 2,357 | 6,535 |
| 4 interviews |  |  |  | 2,776 | 2,776 |
| Total | 15,613 | 16,563 | 9,679 | 7,107 | 48,962 |

Note: The table does not include 15,782 participants in 2018.
